# Does partnership predict mortality? Evidence from a twin fixed effects study design

**DOI:** 10.1016/j.ssmph.2025.101805

**Published:** 2025-04-21

**Authors:** Øyvind Nicolay Wiborg, Alexi Gugushvili

**Affiliations:** a Department of Sociology and Human Geography, University of Oslo, Norway; b DIW-Berlin, Germany

## Abstract

This study examines the association between partnership status and mortality, addressing methodological concerns such as selection effects and unobserved heterogeneity. Utilizing Norwegian administrative data on birth cohorts from 1955 to 1975, we analyze mortality outcomes using Kaplan-Meier survival analyses and Cox proportional hazards regression models. The dataset includes over 1.2 million individuals and distinguishes between partnered and non-partnered individuals. While results for the general population show that non-partnered individuals face significantly higher mortality risks, with hazard ratios of 1.59 for men and 1.47 for women, twin fixed effects models reveal no significant relationship between partnership status and mortality. This finding suggests that much of the observed association may be due to shared genetic and environmental factors rather than a direct causal effect of partnership status. By leveraging the twin fixed effects research design, this study provides a robust test of the partnership-mortality hypothesis, highlighting the importance of controlling for unobserved heterogeneity in observational research. Our results underscore the complexity of the partnership-health nexus and call for caution in interpreting simple associations as evidence of causality.

## Highlights

• Tests the hypothesis that partnered people have lower mortality than those who are unpartnered.
• Twin fixed effects with Cox models offer a robust test, adjusting for unobserved individual differences.
• Link between partnership and mortality may reflect shared genes or environment, not a causal effect.
• Standard models show higher mortality when unpartnered, but twin models find no significant link.

## 1 Introduction

The relationship between partnership status and health outcomes has been a focal point of public health research for decades. Empirical evidence consistently indicates that individuals in committed relationships, such as marriage or cohabitation, experience better health and lower mortality rates compared to their unpartnered counterparts (Kalmijn, 2017; Kiecolt-Glaser & Newton, 2001; Koball et al. 2010; Leung et al. 2022; Wang et al. 2020). This phenomenon, often referred to as the "marriage protection effect," suggests that being in a committed relationship confers health benefits that extend longevity, offering protective effects across physical, mental, and cognitive domains.

Stable partnerships, particularly marriage, have been linked to reduced risks of chronic diseases, improved mental well-being, and lower levels of frailty (Grundström et al. 2021; Jia & Lubetkin, 2020; Syse & Lyngstad, 2017; Wang et al. 2023). In contrast, being single or never married is associated with poorer health outcomes, including higher rates of depression, lower self-esteem, and shorter life expectancy, especially among men and older adults (Grundström et al. 2021; Jennings et al. 2022; Jia & Lubetkin, 2020; Williams et al. 2017). Marital disruptions, such as divorce or widowhood, further exacerbate these risks, primarily due to the loss of social, emotional, and economic support, with effects particularly pronounced among men, younger adults, and individuals with lower socioeconomic status (An et al. 2023; Choi et al. 2022; Næss et al. 2021; Wang et al. 2023). This evidence underscores the important role of partnership status in shaping health outcomes and highlights the need to understand the underlying mechanisms driving these associations.

In addition to the broad evidence linking partnerships to various dimensions of health, numerous studies have examined the specific association between relationship status and mortality. Research consistently finds that unmarried individuals face elevated all-cause and cause-specific mortality risks compared to their married or cohabiting counterparts, including from cardiovascular disease, cancer, and external causes (Kaplan, 2006; Manfredini et al. 2017; Wong et al. 2018). These mortality differences are especially pronounced among men and have been observed across diverse contexts and study designs. Meta-analytical reviews confirm that the mortality gap persists even after adjusting for confounding factors, suggesting that relationship status remains a robust predictor of survival (Rendall et al. 2011; Robards et al. 2012). These findings provide important context for the current study's focus on mortality as a central outcome and reinforce the need to understand whether this association reflects a causal relationship or selection processes.

Several mechanisms have been proposed to explain how partnership status influences health outcomes. Beyond the higher socioeconomic status often associated with married individuals, mechanisms such as emotional support, social attachment, and health behavior regulation are thought to explain the relationship between partnership status and mortality (Pietromonaco et al. 2013; Pietromonaco & Collins, 2017; Umberson et al. 2018; Wilson and Novak, 2022). Emotional support from a partner can mitigate stress and its adverse effects on health, while social attachment provides a sense of belonging and purpose. Partners often play a role in regulating health behaviors, encouraging positive activities such as regular exercise and balanced diets, and discouraging detrimental habits like smoking and excessive alcohol consumption. Additionally, the pooling of economic resources in partnerships can enhance access to healthcare services and healthier living conditions (Bolger et al. 2000; Jackson et al. 2015; Robles et al. 2014; Waite & Gallagher, 2001).

Nonetheless, the association between partnership status and health raises questions about causation and selection. The social causation hypothesis posits that being in a partnership directly enhances health through mechanisms such as emotional support, social attachment, and health behavior regulation. Alternatively, the health selection hypothesis suggests that healthier individuals are more likely to marry or cohabitate, indicating that health status influences partnership status rather than the reverse (Franke & Kulu, 2018; Hanson et al. 2014; Horn et al. 2013; Joung et al. 1998). Disentangling these hypotheses is complex, as partnered individuals may differ systematically from non-partnered individuals in terms of social background, personality traits, and family upbringing, factors that independently affect health and survival outcomes (Beam et al. 2018; Jerskey et al. 2010).

Longitudinal studies and genetically informed research designs, such as twin studies, can provide more robust evidence for the causal impact of partnership on health by controlling for unaccounted selection effects. However, the generalizability of these findings across diverse cultural and socioeconomic contexts remains a challenge, as partnership dynamics and associated health benefits vary widely across populations. The observed benefits of partnerships may reflect preexisting advantages rather than causal effects. These unmeasured individual characteristics, such as personality traits and family upbringing, complicate the interpretation of findings and underscore the need for research designs that can better isolate causal effects from selection biases.

Gender differences further complicate the relationship between partnership status and health. Research has consistently shown that men benefit more from committed relationships than women (Chung & Kim, 2015; Rendall et al. 2011; Wójcik et al. 2021). Unmarried men face higher risks of mortality, particularly from cardiovascular diseases, compared to married men (Manfredini et al. 2017; Raparelli et al. 2018; Rendall et al. 2011). The health benefits for men in partnerships may stem from increased social support and health monitoring provided by their partners. While women also experience health benefits from partnerships, the effects are generally less pronounced. This difference may be due to women's broader social networks and differing social roles, which provide support beyond that of a partner. Additionally, the quality of the marital relationship plays an important role in women's health outcomes, with high-quality relationships providing significant benefits and low-quality ones potentially leading to adverse effects (Burman & Margolin, 1992; Robles et al. 2014).

Based on the discussion above, in this study, we ask the following overarching question: *Does partnership status influence mortality, or is the observed association driven by selection effects?* While extensive literature highlights the benefits of partnerships for health, much remains unknown about the underlying causal mechanisms due to unobserved heterogeneity. Our research, leveraging Norwegian general population and twin data, offers a unique opportunity to control for genetic and early-life environmental factors, providing greater clarity on this complex relationship. This study is important not only for its methodological contribution but also for its implications for social policies aimed at supporting individuals across different partnership statuses. Our two specific research questions are: *(1) Does partnership status predict mortality when controlling for genetic and environmental factors? (2) How do these associations differ between genders?* Our main hypothesis is that while general population analysis might show a significant association between partnership status and mortality, this relationship attenuates or disappears when controlling for unobserved heterogeneity using twin fixed effects models.

## 2 Methods

### 2.1 Data

This study utilizes Norwegian administrative register data, which offers comprehensive coverage of the entire population. This data is made available from Statistics Norway through the HistClass project at the 10.13039/501100005366University of Oslo (grant number 578502). The use of data is approved by SIKT, the Norwegian Agency for Shared Services in Education and Research. The dataset integrates individual- and family-level information from national censuses, tax records, educational registers, and mortality statistics, enabling a detailed investigation of the relationship between partnership status and mortality. The data contains information on birth cohorts from 1955 to 2000, but the analysis is restricted to individuals born between 1955 and 1975 due to data availability on partnership history and other covariates. As we observe individuals up to 2017, the younger birth cohorts are right censored (see for example Fig. S2). The final analytical selection consists of 621,260 men and 583,922 women. Gender is classified according to the binary legal categorization used in official Norwegian records.

The primary outcome variable is mortality, measured as the occurrence of death and its timing relative to the baseline at age 37. Mortality data are linked to individual records using unique personal identifiers, ensuring accurate longitudinal tracking of survival status. The number of individuals with an immigrant background is relatively low in this analytical selection. One reason is that the last step of our analyses (the sibling/twin fixed effects analyses) requires that there is a pointer to parents. Individuals with immigrant background tend to lack that information. Another reason is that many of our baseline variables are measured at the age of 37, which excludes immigrants arriving after that age.

### 2.2 Independent variables

The key independent variable is partnership status, classified into three categories: Partnered individuals (married or cohabiting), divorced/separated/widowed, and non-partnered individuals (unmarried individuals). This categorization reflects the social and economic significance of cohabitation in Scandinavian contexts, where cohabiting unions often function similarly to marriages in providing financial, emotional, and social support. The information about partnerships is compiled by Statistics Norway's Population Statistics section, based on registered data from administrative population registers on civil status (marriage, cohabitation, divorce, separation, and children) and individual residency units.

Control variables include migration background, education, and earnings, measured at baseline (age 37), and the number of children, birth order and number of siblings. The migration variable distinguishes between individuals born in Norway and those with an immigrant background. Educational attainment is categorized into (1) primary school and lower secondary, (2) upper secondary, and tertiary education (BA = 3, MA/PhD = 4). The earnings are measured as an average of two earnings years (at age 37–38) to reduce the impact of transitory fluctuations. And earnings are also adjusted for inflation and are ranked within each income year as a cumulative density rank function variable in order to ensure comparability of the position in the earnings distribution for different birth cohorts. The earnings variable is constrained between zero (lowest part of the earnings distribution) and one (highest part of the earnings distribution). Sibling fixed effects and twin fixed effects are included in separate analysis to account for shared familial and genetic influences. This allows us to distinguish between the effects of partnership status per se and those driven by unmeasured confounders within families. Table 1 demonstrates descriptive statistics of variables employed in the present study.

**Table 1:** Descriptive Statistics at age 37/38.

	General population			Twin population		
	Men	Women	Total	Men	Women	Total
N	621,820 (51.6 %)	584,232 (48.4 %)	1,206,052 (100.0 %)	11,536 (50.9 %)	11,148 (49.1 %)	22,684 (100.0 %)
Partnership status						
Non-partnered	195,701 (31.5 %)	152,091 (26.0 %)	347,792 (28.8 %)	3926 (34.0 %)	3062 (27.5 %)	6988 (30.8 %)
Divorced/Separated/Widowed	16,819 (2.7 %)	22,478 (3.8 %)	39,297 (3.3 %)	291 (2.5 %)	384 (3.4 %)	675 (3.0 %)
Partnered	409,300 (65.8 %)	409,663 (70.1 %)	818,963 (67.9 %)	7319 (63.4 %)	7702 (69.1 %)	15,021 (66.2 %)
Immigration background						
No imm. background	583,670 (93.9 %)	548,057 (93.8 %)	1,131,727 (93.8 %)	10,787 (93.5 %)	10,424 (93.5 %)	21,211 (93.5 %)
Immigrant	5645 (0.9 %)	4821 (0.8 %)	10,466 (0.9 %)	92 (0.8 %)	69 (0.6 %)	161 (0.7 %)
Descendant	1206 (0.2 %)	1154 (0.2 %)	2360 (0.2 %)	34 (0.3 %)	16 (0.1 %)	50 (0.2 %)
Other	31,299 (5.0 %)	30,200 (5.2 %)	61,499 (5.1 %)	623 (5.4 %)	639 (5.7 %)	1262 (5.6 %)
Education Level						
Lower Secondary	221,813 (35.7 %)	227,825 (39.0 %)	449,638 (37.3 %)	4306 (37.3 %)	4650 (41.7 %)	8956 (39.5 %)
Upper Secondary	227,108 (36.5 %)	158,022 (27.0 %)	385,130 (31.9 %)	4150 (36.0 %)	2946 (26.4 %)	7096 (31.3 %)
Lower Tertiary (BA)	123,861 (19.9 %)	167,360 (28.6 %)	291,221 (24.1 %)	2124 (18.4 %)	2989 (26.8 %)	5113 (22.5 %)
Tertiary, high (MA/PhD)	49,038 (7.9 %)	31,025 (5.3 %)	80,063 (6.6 %)	956 (8.3 %)	563 (5.1 %)	1519 (6.7 %)
Birthyear	1965.150 (5.972)	1965.263 (5.971)	1965.205 (5.972)	1964.836 (5.938)	1964.974 (5.914)	1964.904 (5.926)
Number of children	1.927 (1.238)	2.061 (1.100)	1.992 (1.175)	1.896 (1.270)	2.018 (1.135)	1.956 (1.207)
Birth order	2.090 (1.201)	2.093 (1.200)	2.091 (1.201)	3.305 (1.290)	3.292 (1.289)	3.299 (1.290)
Number of siblings	2.156 (1.392)	2.160 (1.388)	2.158 (1.390)	2.904 (1.464)	2.910 (1.451)	2.907 (1.457)
Earnings (100K NOK)	5.088 (3.685)	3.275 (1.886)	4.210 (3.090)	4.998 (2.824)	3.243 (1.846)	4.136 (2.549)
Earnings (cdr)	0.636 (0.263)	0.388 (0.247)	0.516 (0.284)	0.636 (0.260)	0.387 (0.246)	0.514 (0.282)

### 2.3 Statistical analysis

We apply an event history analytical approach using Kaplan-Meier survival analysis and Cox proportional hazards regression models to examine how partnership status is associated with mortality risk. The Cox models estimate hazard ratios for mortality, controlling for key covariates and fixed effects for having the same mother among siblings and twins. We rely on the standard Cox proportional hazard model and its modified fixed effects version (Allison 2009). In our setup, the modified fixed effects Cox regression equation can be presented as:

$${\text{logh}}_{\text{ik}}\left(t\right)=\mu_{i}\left(t-t_{\text{ik}-1}\right)+\beta_{1}*{\left(\text{partnership}\;\text{status}\right)}_{\text{ik}}+\beta_{2}x_{\text{ik}}$$

*Partnership status*_*ik*_ is the main explanatory variable, while *x*_*ik*_ represents a vector for the control variables in the model. Both may vary across families (*i*) and siblings (*k*). *β*_*1*_ and *β*_*2*_ represents a row vector of their corresponding coefficients. *t*_*i*(*k*-1)_ denotes the time of the (*k* − 1)^th^ sibling. In a regular Cox model, the term *μ*(·) represents an unspecified function of the duration since the most recent event, and in a regular Cox model, it is assumed to be the same for all the individuals and families. In our modified fixed effects model, the term has a prefix for each family *μ*_*i*_(·). This means that each family has their own hazard function, which absorbs everything shared for each family member. For the twin fixed effects analysis, we further constrain the family fixed effects to siblings born in the same year, ensuring that comparisons are only made within twin pairs while still controlling for shared family-level factors.

To address selection bias and unobserved heterogeneity, we employ twin fixed effects models, which compare mortality outcomes within twin pairs (Pongou et al. 2017; Royer, 2009). This method isolates the effect of a partnership status while holding constant the unmeasured characteristics that are identical or highly similar within the pair (Lundborg et al. 2016). By leveraging the natural experiment framework, we provide stronger causal inference regarding the relationship between partnership and mortality. Given the potential violation of the proportional hazards assumption, we assess model fit using Schoenfeld residuals, and alternative parametric survival models (such as Weibull and exponential models) are estimated to confirm robustness. While the twin fixed effects design enhances causal interpretation, it also introduces limitations, such as reduced statistical power due to smaller sample sizes within twin pairs. Further, Norway's extensive welfare provisions may attenuate some of the adverse health effects of non-partnered status, limiting direct comparability with studies conducted in different institutional contexts.

## 3 Results

### 3.1 Kaplan-Meier survival analysis

**Fig. 1:**
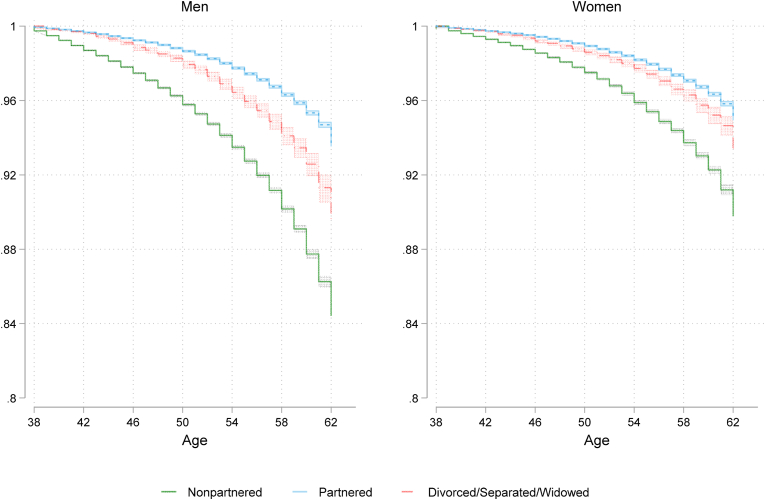
Kaplan-Meier survival analyses by partnership status and gender, from age 38 to 62. The general population consists of individuals born between 1955 and 1975 (N = 621,820 men; N = 584,232 women). Partnered individuals (N = 818,963) include those who are married or cohabiting with common children, while non-partnered individuals (N = 347,792) consist of those who are unmarried, or cohabiting without children (see note in data section). And the third category consists of individuals who are divorced, separated or widowed (N = 39,297).

The Kaplan-Meier survival analysis in Fig. 1 tracks individuals by their partnership status at age 37, following them until age 62. The overall patterns of association between partnership status and mortality are consistent across both genders. By the end of the analytical period, survival rates for partnered men and women are similar, with nearly 95 % of partnered individuals still alive at age 62. Over the 24-year period, approximately 85 % of non-partnered men and 90 % of non-partnered women survive. We can further differentiate the non-partnered category from divorced/separated/widowed individuals and observe that this category of men has a lower likelihood of mortality than the non-partnered individuals. At the same time, among women, there is almost no difference between partnered and divorced/separated/widowed individuals.

### 3.2 Cox proportional hazard models

**Fig. 2:**
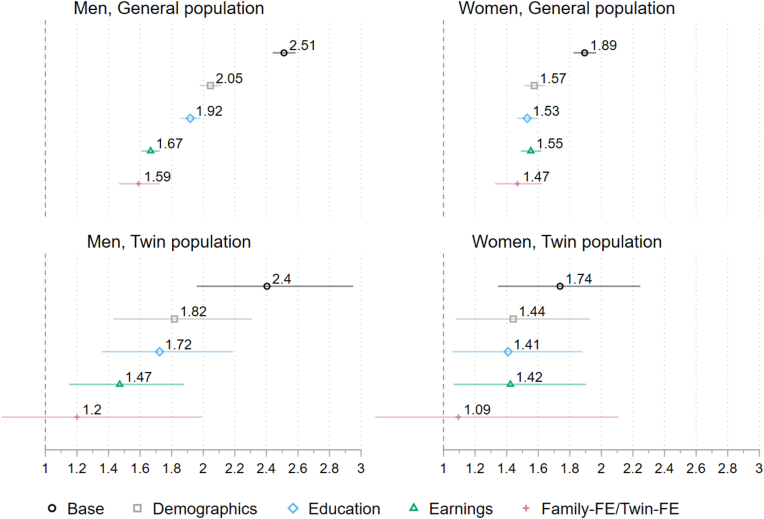
Cox regression models for men (N = 621,820) and women (N = 584,232), showing hazard ratios for mortality based on partnership status (non-partnered vs. partnered). The results for the divorced/separated/widowed category are shown in Fig. S1. Stepwise controls are included for potential confounders, such as demographics (immigrant background, number children, birth order, number of siblings), education level, earnings, and sibling-FE (shared family background in the sibling fixed effects analyses). Based on models in Supplement Table S2. 95 % Confidence intervals.

Findings from the Cox proportional hazards regression models for the general population, shown in Fig. 2, confirm that being non-partnered in comparison to being partnered is associated with higher mortality rates, with hazard ratios of 1.59 for men and 1.47 for women, even after accounting for observable confounders and sibling-fixed effects. Nonetheless, when we run models with twin populations of men and women that account for twin fixed effects, the effect of being in a partnership becomes insignificant. In other words, among twin men and women, partnership status does not predict mortality outcomes. The full results are shown in Tables S1 and S2. Fig. S1 and Table S2 in the Supplementary Materials also show divorced/separated/widowed individuals’ mortality outcomes compared to partnered individuals. The main findings are essentially similar – partnered men and women do not have better mortality outcomes when the effects are estimated using twin fixed effects models.

### 3.3 Additional analysis

We also conducted additional analyses to examine whether the association between partnership status and mortality varies across subgroups beyond gender. Fig. S2 presents the results stratified by educational attainment, showing that the association is stronger among individuals with lower education compared to those with higher education. A similar pattern is observed when examining personal wealth, with poorer individuals who are not partnered facing particularly higher mortality risks (Gugushvili & Wiborg, 2025). However, after adjusting for control variables and incorporating twin fixed effects in the Cox regression models, these stronger associations for individuals with lower education are no longer evident. Additionally, Fig. S3 presents separate estimates for individuals born before and after 1965, indicating that the investigated association does not differ markedly between older and younger cohorts.

## 4 Discussion

The relationship between partnership status and mortality has been extensively studied, with a consensus that partnered individuals generally exhibit lower mortality rates compared to their non-partnered counterparts. This protective effect of partnerships is often attributed to various factors, including emotional support, shared economic resources, and health-promoting behaviors encouraged within partnerships (Gardner & Oswald, 2004; Lillard & Panis, 1996; Monden, 2007; Rendall et al. 2011; Robards et al. 2012). For instance, a meta-analytic review conducted by Holt-Lunstad et al. (2010) demonstrates that strong social relationships, including marital and cohabiting partnerships, are associated with a 50 % increased likelihood of survival, underscoring the significant impact of social ties on longevity. However, the underlying mechanisms of the partnership's effect on health remain a subject of debate, particularly concerning the roles of selection and causation. The selection hypothesis posits that healthier individuals are more likely to enter and maintain partnerships, thereby confounding the observed protective effects. In contrast, the causation hypothesis suggests that partnerships directly confer health benefits through mechanisms such as, among others, social support and improved economic stability (van den Berg & Gupta, 2015; Franke & Kulu, 2018; Hanson et al. 2014; Horn et al. 2013).

Our study contributes to this debate by employing a twin fixed effects research design, a methodological approach that allows for the control of unobserved genetic and shared environmental factors. By analyzing data from twin pairs in Norway, we can account for inherent genetic predispositions and early-life environmental influences that traditional observational and longitudinal studies might overlook. This design enables a more accurate estimation of the causal impact of partnership status on mortality. Our findings indicate that while non-partnered individuals exhibit higher mortality risks in the general population, these associations lose statistical significance within twin pairs. This attenuation suggests that the observed protective effect of partnerships may be largely attributable to selection factors rather than a direct causal relationship. Specifically, twins share numerous unmeasured characteristics, such as genetic predispositions to certain health conditions and early-life environmental exposures, which can influence both partnership formation and mortality risk. By controlling for these factors, our study provides evidence that challenges the notion of a direct protective effect of partnership on mortality.

These results align with some previous research that has questioned the causal nature of the partnership-mortality link. For example, a study by Franke and Kulu (2018) found that while partnered individuals had lower mortality rates, the differences diminished after accounting for selection effects related to health and socioeconomic status. Similarly, Requena and Reher (2021) highlighted the importance of considering selection biases when interpreting the protective effects of marriage on health outcomes. Our study extends the existing literature by highlighting the significance of genetic and early-life environmental factors in shaping both partnership trajectories and health outcomes. The use of twin data provides a unique opportunity to disentangle these complex relationships, offering insights that are not attainable through studies focusing solely on non-twin populations. Still, possible causal effects of partnerships on mortality could vary by socio-economic subpopulations, which should be scrutinized further. One recent study shows that personal wealth might interact with partnership and gender with regard to mortality in Norway (Gugushvili & Wiborg, 2025).

Our findings also raise questions about the mechanisms often proposed to explain the protective effects of partnerships, such as emotional support, social integration, and health behavior regulation. While these mechanisms may still play a role, the twin analysis suggests that their contribution might be less pronounced or that their effects are mediated by other factors shared by twins, such as personality traits or family upbringing. This calls for further research to disentangle the interplay between individual, relational, and contextual factors in shaping health outcomes.

Previous research has highlighted gender differences in the health benefits of partnership, with some studies suggesting that men derive greater protective effects than women due to differences in social support structures and health behaviors. However, our results do not indicate that partnership status significantly affects mortality differently for men and women. In the general population models, non-partnered men exhibited slightly higher mortality risks compared to non-partnered women, consistent with prior findings. Once genetic and early-life environmental factors were accounted for in twin fixed effects models, these differences were no longer statistically significant. This suggests again that the observed gender disparities in partnership effects may largely reflect selection processes rather than a true causal relationship. The findings challenge the assumption that partnership status exerts a fundamentally different impact on men's and women's mortality risks and underscore the need for future research to explore the role of relationship quality, social networks, and broader life-course conditions in shaping these associations.

Beyond its methodological contribution, our study also offers implications for social policy. The absence of a significant causal effect of partnership status on mortality in our twin fixed effects models indicates that policies aimed solely at promoting formal partnerships may have limited success in improving population-level health outcomes. Instead, greater emphasis should be placed on addressing the underlying social, economic, and psychological conditions that influence both relationship formation and mortality risk. Policymakers should consider strengthening support for non-partnered individuals, including through targeted mental health services, community-building initiatives, and economic assistance programs, particularly in contexts where social safety nets are weaker than in Norway. Understanding that the health advantages associated with partnerships may reflect deeper social selection mechanisms helps shift attention toward more inclusive and effective health and welfare policies.

### 4.1 Limitations

Despite the strengths of our approach, certain limitations warrant discussion. Our inability to account for informal romantic relationships, such as dating, may overlook additional sources of social and emotional support that could influence health outcomes. Previous studies have demonstrated that the quality of intimate relationships, regardless of formal status, plays an important role in health and longevity. For instance, research has shown that high-quality intimate relationships positively impact physical health, including cardiovascular, immune, and endocrine systems, and are associated with lower mortality risk (Robles et al. 2014). Therefore, future research should aim to incorporate a broader spectrum of relationship types to fully understand the dynamics between social connections and mortality. Additionally, while twin studies offer robust control for shared genetic and environmental factors, they may not fully capture individual-level variables such as current mental health status or life-course socioeconomic changes. These factors could independently affect both partnership status and mortality risk, suggesting avenues for future research to incorporate dynamic measures of individual experiences and conditions.

While our use of Norwegian twin data offers a unique advantage in controlling for unobserved genetic and environmental heterogeneity, the broader generalizability of our findings might be limited. The dataset primarily represents a relatively homogeneous population in terms of racial/ethnic diversity, and the twin population may not fully reflect the broader population's socio-economic heterogeneity. Future research should aim to replicate these findings using more diverse datasets, including populations from different socio-economic strata and racial/ethnic groups, to enhance the external validity of the results. Additionally, while twin fixed effects models control for shared genetic and early-life environmental factors, they may not capture later-life socio-economic changes or environmental exposures that can affect both partnership status and mortality. Also, epigenetic modifications, which can be influenced by environmental exposures, lifestyle choices, and randomness, may alter gene expression in ways that affect siblings' health outcomes differently, even among identical twins. Especially if differences in environmental exposures accumulate over time, this could introduce heterogeneity in the estimated effects. So, while our fixed-effects approach eliminates time-invariant confounders, it does not account for these dynamic, individual-level variations that could arise between siblings and even between twins. If these time-changing factors are correlated with partnership status and mortality, they could potentially bias the results. Thus, future research could for example incorporate additional controls for observable lifestyle differences.

Methodologically, this study illustrates the value of twin fixed effects models in addressing issues of unobserved heterogeneity and selection bias. However, these models also have limitations, such as reduced statistical power due to smaller sample sizes and the inability to account for within-pair differences in shared environmental exposures over time. Additionally, while twins provide a unique natural experiment, they may not be fully representative of the broader population, particularly in terms of partnership dynamics and mortality risks. Another limitation of our study is the relatively large confidence intervals in the twin fixed effects models, reflecting reduced precision compared to the general population analysis. This is due to the smaller sample size and limited variation in partnership status within twin pairs. Nonetheless, the twin design remains valuable for addressing unobserved genetic and early-life environmental confounders that traditional observational studies cannot fully capture.

Further, comparing our effect sizes with prior literature is challenging, as most studies rely on general population samples and regression techniques that do not account for familial confounding. The notable reduction in effect sizes in our twin models suggests that selection effects play a key role in the observed association between partnership status and mortality. The strong institutional support in Norway may buffer some of the negative health consequences of being non-partnered, making direct comparisons with studies from different welfare contexts difficult. Future research should examine these associations across diverse institutional settings and expand twin-based approaches to improve external validity. Despite limitations in precision, our findings highlight the importance of accounting for selection effects in studies on social relationships and mortality, reinforcing the value of twin fixed effects models in distinguishing causation from selection bias.

Lastly, while our study provides valuable insights into the relationship between partnership status and mortality, it does not capture changes in partnership status over time or the length of partnerships. Prior research suggests that both partnership stability and transitions, such as divorce or widowhood, can have significant health implications (Hughes & Waite, 2009; Robles et al. 2014). Future studies should explore the effects of relationship duration and transitions using longitudinal data with repeated measures of partnership status, which would allow for a more nuanced understanding of how different partnership trajectories influence mortality risk.

## 5 Conclusion

This study highlights the importance of critically evaluating the relationship between partnership status and mortality rather than assuming it to be purely causal. While partnerships may indeed offer benefits for health and longevity, our findings highlight the need for careful consideration of confounding factors and the use of robust analytical techniques to understand the complexities of this association better. Future research should aim to replicate these findings in other contexts and explore additional mechanisms that may underlie the observed patterns.

## CRediT authorship contribution statement

**Øyvind Nicolay Wiborg:** Writing – original draft, Visualization, Methodology, Formal analysis, Data curation, Conceptualization. **Alexi Gugushvili:** Writing – original draft, Conceptualization.

## Ethics declaration

The use of register data is approved by SIKT, the Norwegian Agency for Shared Services in Education and Research.

## Declaration of competing interests

The authors have no conflicts of interest relevant to this article to disclose.

This work was supported by the Research Council of Norway (HistClass project at University of Oslo, grant number 578502)*.* The sponsor did not play any role in the study design; in the analysis and interpretation of data; in the writing of the report; and in the decision to submit the article for publication.

## Data Availability

The authors do not have permission to share data.
