# Supplementary material for: Does partnership predict mortality? Evidence from a twin fixed effects study design
Source: SSM Popul Health. 2025 Apr 21;30:101805. doi: 10.1016/j.ssmph.2025.101805 (PMC12098163; doi:10.1016/j.ssmph.2025.101805)
Supplement: Multimedia component 1 [file mmc1.docx]

Table S1: Cox regression models underlying the analyses of the General population in Figure 2.

Table S2: Cox regression models underlying the analyses of the Twin population in Figure 2.

Figure S1: Cox regression models for men (N= 621,820) and women (N=584,232), showing hazard ratios for mortality based on partnership status (Divorced/Separated/widowed vs. partnered). Stepwise controls are included for potential confounders, such as immigrant background, education level, earnings, and sibling-FE (shared family background in the sibling fixed effects analyses). Based on models in Supplement Table S2. The category of Divorced/Separated/widowed is very small the twins, see Table 1, thus leading to large standard errors and wide 95%-Confidence Intervals.

Figure S2: Kaplan-Meyer survival rates broken down by educational levels (1=primary school/lower secondary, 2=upper secondary, 3=BA, 4=MA/PhD). 95%-CI.

Figure S3: Kaplan-Meyer survival rates broken down by selected birth cohorts before and after 1965. General and Twin populations. 95%-CI.
